# Islatravir Is Not Expected to Be a Victim or Perpetrator of Drug-Drug Interactions via Major Drug-Metabolizing Enzymes or Transporters

**DOI:** 10.3390/v13081566

**Published:** 2021-08-07

**Authors:** Kelly Bleasby, Robert Houle, Michael Hafey, Meihong Lin, Jingjing Guo, Bing Lu, Rosa I. Sanchez, Kerry L. Fillgrove

**Affiliations:** Merck & Co., Inc., Kenilworth, NJ 07033, USA; Kelly_Bleasby@merck.com (K.B.); robert_houle@merck.com (R.H.); michael_hafey@merck.com (M.H.); meihong.lin@merck.com (M.L.); jingjing.guo@merck.com (J.G.); bing_lu@merck.com (B.L.); rosa_sanchez@merck.com (R.I.S.)

**Keywords:** 4′-ethynyl-2-fluoro-2′-deoxyadenosine (EFdA), islatravir, MK-8591, cytochrome p450, drug–drug interaction, drug transporters, HIV-1, nucleoside reverse transcriptase translocation inhibitor, concomitant medication, antiretroviral agents

## Abstract

Islatravir (MK-8591) is a nucleoside reverse transcriptase translocation inhibitor in development for the treatment and prevention of HIV-1. The potential for islatravir to interact with commonly co-prescribed medications was studied in vitro. Elimination of islatravir is expected to be balanced between adenosine deaminase–mediated metabolism and renal excretion. Islatravir did not inhibit uridine diphosphate glucuronosyltransferase 1A1 or cytochrome p450 (CYP) enzymes CYP1A2, 2B6, 2C8, 2C9, 2C19, 2D6, or 3A4, nor did it induce CYP1A2, 2B6, or 3A4. Islatravir did not inhibit hepatic transporters organic anion transporting polypeptide (OATP) 1B1, OATP1B3, organic cation transporter (OCT) 1, bile salt export pump (BSEP), multidrug resistance-associated protein (MRP) 2, MRP3, or MRP4. Islatravir was neither a substrate nor a significant inhibitor of renal transporters organic anion transporter (OAT) 1, OAT3, OCT2, multidrug and toxin extrusion protein (MATE) 1, or MATE2K. Islatravir did not significantly inhibit P-glycoprotein and breast cancer resistance protein (BCRP); however, it was a substrate of BCRP, which is not expected to be of clinical significance. These findings suggest islatravir is unlikely to be the victim or perpetrator of drug-drug interactions with commonly co-prescribed medications, including statins, diuretics, anti-diabetic drugs, proton pump inhibitors, anticoagulants, benzodiazepines, and selective serotonin reuptake inhibitors.

## 1. Introduction

HIV-1 can now be managed as a chronic illness with lifelong combination antiretroviral therapy (ART); the life expectancy of people living with HIV (PLWH) is approaching that of people without HIV, particularly in high-income nations [1,2]. Although progress in the treatment of HIV-1 with ART has greatly reduced the morbidity and mortality associated with this infection, substantial disease management challenges remain [2,3,4].

PLWH have an increased risk of accelerated aging and the development of comorbidities [5,6], including diabetes, cardiovascular disease, chronic liver disease, and chronic kidney disease [2,7,8]. Therefore, in addition to ART, PLWH often require medications to treat their comorbidities, such as statins, diuretics, antidiabetic drugs, or benzodiazepines, which can lead to considerable polypharmacy and necessitates consideration of potential drug–drug interactions, adverse events, food restrictions, and complicated administration schedules [9,10,11]. The high frequency of drug interactions seen in PLWH receiving polypharmacy can result in adverse health outcomes and has typically required treatment modification or increased monitoring [12].

Pharmacokinetic drug interactions result from changes in plasma concentrations of a ‘victim’ drug caused by a ‘perpetrator’ drug altering the metabolism or transporter-mediated disposition of the victim drug [13]. An increase in victim drug concentrations usually occurs when metabolism or transporter-dependent elimination of that drug is inhibited by a perpetrator, increasing the risk for accumulation in plasma and tissues, as well as drug-related toxicities. Conversely, when metabolism or transporter-dependent elimination of the victim drug is augmented by the perpetrator drug, concentrations of the victim drug will decrease, which may reduce its efficacy. For antiretroviral agents, the result is suboptimal suppression of HIV, leading to the development of resistance, viral rebound, and increased risk of virus transmission. Characterization of the potential for drug interactions between new antiretroviral agents and established antiretroviral agents with which they may be co-administered, or with common non-HIV medications, is currently stipulated in regulatory agency guidance [14,15,16].

**Figure 1:**
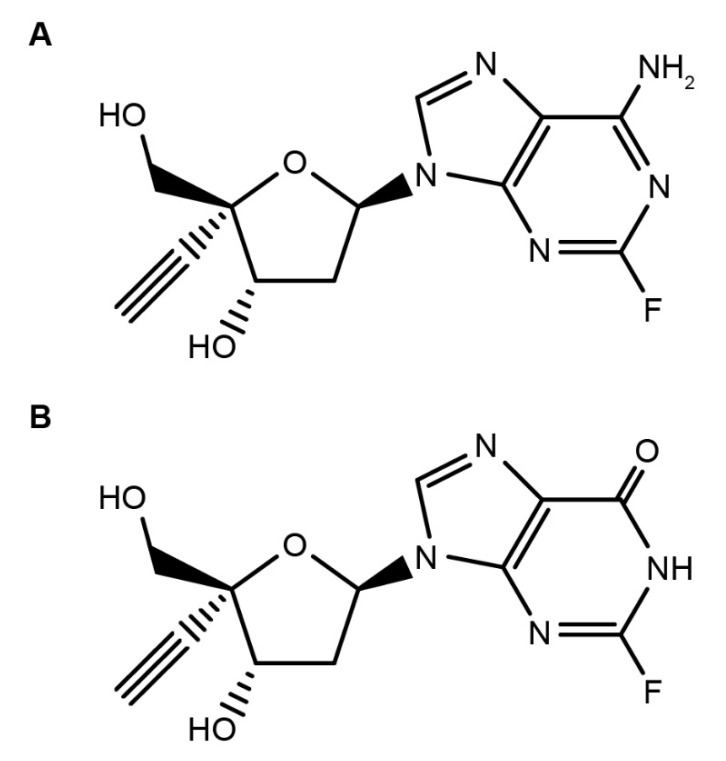
Structure of (**A**) islatravir and (**B**) metabolite M4 4′-ethynyl-2-fluoro-2′-deoxyinosine.

Islatravir (MK-8591) is a nucleoside reverse transcriptase translocation inhibitor (NRTTI) in development for the treatment and prevention of HIV-1 (Figure 1A) [17,18]. Islatravir inhibits reverse transcriptase (RT) by multiple mechanisms of action, including RT translocation inhibition and delayed chain termination through viral DNA structural changes [19,20,21]. Islatravir is being developed to address the need for new antiretroviral agents with favorable safety and tolerability profiles, high potency, and a high barrier to the development of resistance that may also allow for simplification of treatment [22].

Islatravir has a favorable pharmacokinetic profile and is rapidly converted intracellularly by endogenous kinases to its active triphosphate (TP), islatravir-TP, which inhibits RT by multiple mechanisms to suppress HIV-1 replication [18,20,21,23,24,25]. In treatment-naive PLWH, islatravir was rapidly absorbed and plasma exposure was approximately dose proportional after oral administration with similar pharmacokinetics (PK) in adults without HIV. Islatravir-TP had a long intracellular half-life of ~78.5–128 h, in agreement with the viral load reduction maintained for 7 days after a single administration of islatravir at a dose as low as 0.5 mg [26].

In treatment-naïve PLWH, islatravir administered orally in daily doses of between 0.5 and 30 mg effectively suppressed viral load for at least 7 days [26]. Islatravir was generally well tolerated in participants with and without HIV across a range of doses [26,27]. Owing to the high potency, high barrier to the development of resistance, and long intracellular half-life of islatravir-TP, islatravir has the potential to be effective in a variety of dosing options and regimens for the treatment and prevention of HIV-1. The combination of islatravir with doravirine, a non-nucleoside reverse transcriptase inhibitor (NNRTI), is currently being evaluated in a comprehensive phase 3 clinical program across diverse groups of PLWH, including treatment-naive and treatment-experienced populations (ClinicalTrials.gov ID: NCT04223778, https://clinicaltrials.gov/ct2/show/NCT04223778; NCT04223791, https://clinicaltrials.gov/ct2/show/NCT04223791; NCT04233879, https://clinicaltrials.gov/ct2/show/NCT04233879, accessed on 22 July 2021). In heavily treatment experienced PLWH who are failing their current ART regimen, islatravir and doravirine will be administered in combination with optimized background ART (ClinicalTrials.gov ID: NCT04233216). Islatravir is also being investigated for HIV-1 pre-exposure prophylaxis in at-risk individuals as a once-monthly oral regimen and a once-yearly implant [28,29].

**Figure 2:**
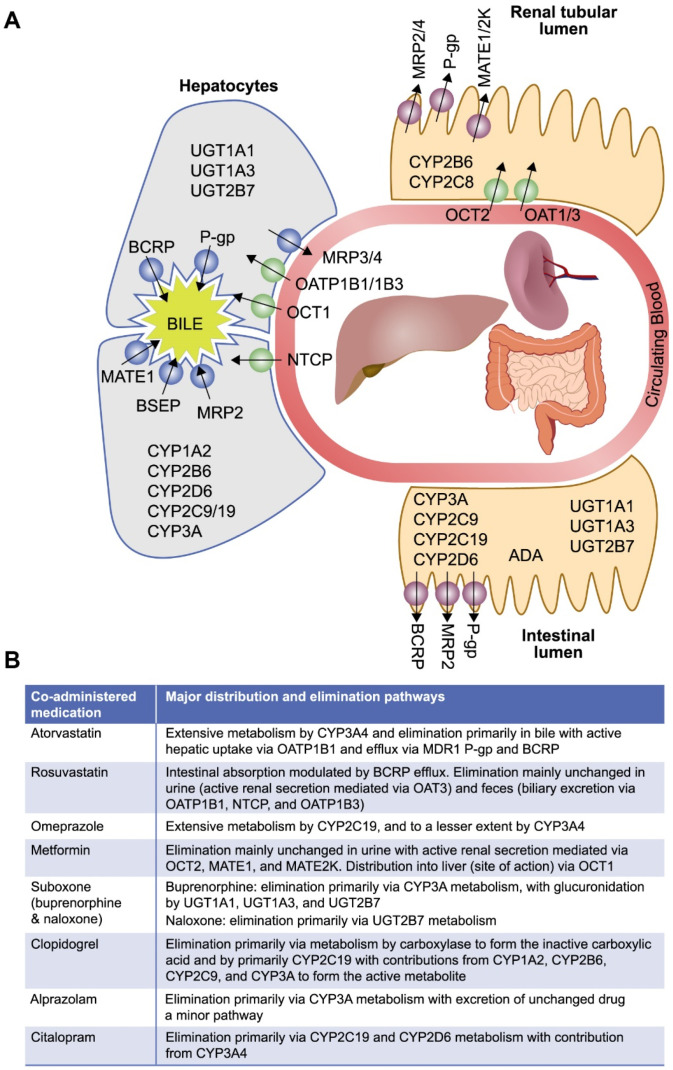
Key elimination pathways of commonly co-prescribed medications * [34,35,36,37,38,39,40,41,42,43,44,45,46,47,48,49,50,51,52,53]. (**A**) Location of drug-metabolizing enzymes and transporters of interest. (**B**) Elimination and distribution pathways for co-administered medications with islatravir *. ADA, adenosine deaminase; BCRP, breast cancer resistance protein; BSEP, bile salt export pump; CYP, cytochrome P450; MATE, multidrug and toxin extrusion protein; MDR1 P-gp, multidrug resistance protein 1 P-glycoprotein; MRP, multidrug resistance-associated protein; OAT, organic anion transporter; OATP, organic anion transporting polypeptide; OCT, organic cation transporter; NTCP, sodium taurocholate co-transporting polypeptide; UGT, uridine diphosphate glucuronosyltransferase. * Commonly prescribed co-medications taken from European Medicines Agency scientific advice on metabolic and elimination pathways for key medications expected to be taken concomitantly with islatravir.

Herein we describe in vitro studies conducted to characterize the distribution and metabolism of islatravir and to establish the potential for islatravir to interact with other drugs via major drug-metabolizing enzymes, and with transporters involved in clinically relevant drug interactions. The selection of drug-metabolizing enzymes and transporters in these studies was based upon the known metabolic properties and elimination pathways for islatravir and commonly prescribed concomitant medications in PLWH, aligning with regulatory guidance on the evaluation of the drug interaction potential of new molecular entities [14,16,30]. The selected drug-metabolizing enzymes and transporters involved in clinically relevant drug interactions include cytochrome P450 (CYP) enzymes and uridine diphosphate glucuronosyltransferase 1A1 (UGT1A1); the hepatic uptake transporters organic anion transporting polypeptide (OATP) 1B1, OATP1B3, and organic cation transporter (OCT) 1; the renal uptake transporters organic anion transporter (OAT)1, OAT3, and OCT2; the efflux transporters multidrug and toxin extrusion protein (MATE) 1 and MATE2K; and the widely expressed efflux transporters multidrug resistance protein 1 P-glycoprotein (MDR1 P-gp) and breast cancer resistance protein (BCRP) [14,15,16,31,32]. The bile salt export pump (BSEP) and multidrug resistance-associated protein (MRP) 2, MRP3, and MRP4 were also investigated due to their association with drug-induced liver injury [33]. The locations of these selected drug-metabolizing enzymes and transporters of clinical interest, along with the main elimination pathways of commonly prescribed medications in PLWH, are illustrated in Figure 2.

## 2. Materials and Methods

### 2.1. Islatravir Distribution in Plasma

Islatravir plasma protein binding was determined as previously described by equilibrium dialysis [54]. Briefly, 0.1, 1, and 10 µM islatravir was added to human, mouse, rat, rabbit, or monkey plasma and dialyzed against an equal volume of phosphate-buffered saline (pH 7.4) at 37 °C under 10% CO_2_, for 24 h. Samples were extracted with the addition of acetonitrile, vortex-mixed, and centrifuged. The resulting supernatants were analyzed by liquid chromatography with tandem mass spectrometry (LC-MS/MS). The unbound fraction in plasma was calculated as Fraction_unbound_ = islatravir concentration in buffer chamber/islatravir concentration in plasma chamber.

Distribution of islatravir between red blood cells and plasma in human blood was determined at select concentrations ranging from 0.01 to 10 µM. Islatravir was added to aliquots of blood and incubated under 5% CO_2_ for 60 min at 37 °C, followed by separation of the red blood cells from the plasma via centrifugation. To assess its initial whole blood concentration, islatravir was added to aliquots of plasma and incubated under 5% CO_2_ for 60 min at 37 °C to serve as a surrogate for whole blood. Samples were extracted with the addition of acetonitrile, vortex-mixed, and centrifuged. The resulting supernatants were analyzed by LC-MS/MS. The blood to plasma ratio (B:P) was calculated as B:P = islatravir concentration in whole blood/islatravir concentration in plasma separated from blood.

### 2.2. Characterization of Islatravir Metabolism in Intestinal S9 and Metabolism by Human Adenosine Deaminase

The metabolism of islatravir was studied in human intestinal S9 fraction (Xenotech, LLC [Kansas City, KS, USA]). [^3^H]islatravir (5 µM) was incubated at 37 °C for 3 h in 0.1 M potassium phosphate buffer (pH 7.4) containing 1.0 mg/mL S9 protein, 5 mM magnesium chloride, and 1 mM NADPH. Reactions were terminated with a stop solution containing 6 mM EDTA and 6 mM EGTA in 70% methanol. Samples were vortex mixed, centrifuged, and the supernatants were subjected to radiometric LC-MS/MS analysis.

The metabolism of islatravir was also evaluated with recombinant human adenosine deaminase (ADA). Islatravir (50 µM) was incubated at 37 °C for 3 h in 0.05 M HEPES buffer (pH 7.4) containing 1 unit/mL of recombinant human ADA (Novus Biologicals, LLC [Centennial, CO, USA]). Reactions were terminated by the addition of acetonitrile, and the samples were vortex-mixed and centrifuged, and the supernatants were subjected to LC-MS/MS analysis.

Enzyme kinetics were evaluated using increasing concentrations of islatravir incubated with recombinant human ADA, pre-incubated in potassium phosphate buffer for 10 min at 37 °C. Reactions were initiated by the addition of islatravir for 15 min and terminated by acetonitrile:methanol containing stable isotope-labeled islatravir ([^13^C,^15^N_3_]ISL). Samples were then vortex-mixed and centrifuged, and the resulting supernatants were then diluted in water containing 0.1% propionic acid and 0.5% dimethyl sulfoxide. M4 formation was quantified by LC-MS/MS analysis using an authentic M4 standard.

### 2.3. Characterization of Renal Clearance in Animal Models

Male CD-1 mice (*n* = 15), male Wistar-Hannover rats (*n* = 6), female Dutch Belted rabbits (*n* = 3), and rhesus monkeys (*n* = 3) were administered 1 mg/kg islatravir intravenously. Blood samples were collected at specified time intervals following dose administration as were urine samples throughout the study period for each animal model; 0–24 h for mice, rats, and monkeys and 0–48 h for rabbits. Islatravir concentrations in plasma and urine were determined by LC-MS/MS, following a protein precipitation step. Renal clearance was calculated by dividing the amount of unchanged islatravir excreted into urine over the course of the study by the corresponding area under the plasma-concentration time curve (AUC_0-x_) in plasma. AUC_0-x_ was determined using the linear trapezoidal method for ascending concentrations, and the log trapezoidal method for descending concentrations, and the amount of unchanged islatravir excreted into urine was obtained by multiplying the concentration of islatravir in urine by the volume of urine collected over the specified time interval.

### 2.4. Interaction of Islatravir with Drug-Metabolizing Enzymes: CYP Isoforms and UGT1A1

Reversible CYP inhibition was performed in pooled human liver microsomes incubated at 37 °C in a reaction mixture containing the appropriate CYP probe substrate and islatravir (0.05 to 100 µM except CYP3A4, which was tested to 200 μM), as previously reported [55]. Similarly, the potential for islatravir (0.78–100 µM) to inhibit the UGT1A1-mediated glucuronidation of estradiol was measured in pooled human liver microsomes, as previously described [55]. CYP2C19 S-mephenytoin (30 μM) 4′-hydroxylation and CYP2D6 dextromethorphan (10 μM) O-demethylation were assessed over incubation periods of 20 min and used the control inhibitors benzyl-nirvanol and quinidine, respectively. CYP1A2 phenacetin (100 μM) O-deethylation, CYP2B6 bupropion (180 μM) hydroxylation, CYP2C9 diclofenac (10 μM) 4′-hydroxylation, and CYP3A4 testosterone (50 μM) 6β-hydroxylation were assessed over incubation periods of 10 min, and used the control inhibitors α-naphtholflavone, ticlopidine, sulfaphenazole, and ketoconazole, respectively. CYP2C8 amodiaquine (4 μM) N-deethylation and CYP3A4 midazolam (3 μM) 1′-hydroxylation were assessed over incubation periods of 3 min, and used the control inhibitors montelukast and ketoconazole, respectively.

The time-dependent inhibition of major human CYP isoforms (1A2, 2B6, 2C8, 2C9, 2C19, 2D6, or 3A4) was performed in pooled human liver microsomes at islatravir concentrations of 10 and 50 µM, using selective probe substrates for each CYP as previously described [55]. CYP-specific probe substrates were phenacetin (300 µM; incubation time 20 min) for CYP1A2, efavirenz (30 µM; incubation time 25 min) for CYP2B6, amodiaquine (20 µM; incubation time 4 min) for CYP2C8, diclofenac (50 µM; incubation time 12 min) for CYP2C9, *S*-mephenytoin (225 µM; incubation time 25 min) for CYP2C19, bufuralol (50 µM; incubation time 15 min) for CYP2D6, and testosterone (250 µM; incubation time 10 min) for CYP3A4. Positive control incubations using a CYP isoform-specific time-dependent inhibitor, control incubations without inhibitor (containing 1% *v*/*v* methanol only), and incubations without NADPH in the inactivation reactions were also performed to assess the overall time-dependent inhibition potential of islatravir.

The potential for islatravir to induce CYP1A2, CYP2B6, and CYP3A4 was assessed in cryopreserved hepatocytes from three human donors, after 48 h incubation with islatravir (0.1–20 μM). The method was as previously described [55], with the exception that all solutions were replaced after 24 h of incubation. Positive control inducers rifampicin (10 μM), phenobarbital (1000 μM) or omeprazole (50 μM) were used, and after the 48-h incubation period, whole cell-based CYP3A4, CYP2B6, and CYP1A2 enzyme changes were evaluated using testosterone 6β-hydroxylation, bupropion hydroxylation, and phenacetin O-deethylation, respectively, measured by LC-MS/MS detection. Total RNA was isolated for quantitative polymerase chain reaction analysis of CYP3A4, CYP2B6, and CYP1A2 mRNA expression, as described previously [56]. The overall induction potential of islatravir was then assessed based on regulatory agency recommendations [14,15,16], which state the induction potential of an investigational drug should not be ruled out if increases in CYP enzyme mRNA were more than 20% of the response of the positive control.

### 2.5. Evaluation of Islatravir as a Perpetrator of Drug–Drug Interactions via Transporters

Inhibition of recombinant transporter-mediated uptake of probe substrates was determined in recombinant cell lines; HEK293-OATP1B1, HEK293-OATP1B3, CHO-K1-OCT1, CHO-K1-OCT2, MDCKII-OAT1, MDCKII-OAT3, CHO-K1-MATE1, and MDCKII-MATE2K, as described previously [55,57,58], with some modifications to OATP1B1 and OATP1B3 inhibition assays. For these assays, cryopreserved HEK293-OATP1B1 and HEK293-OATP1B3 cell aliquots, used under license agreement from SOLVO^®^ Biotechnology (Hungary), were thawed, recovered, and re-suspended in 96-well glass-coated plates at a density of 0.125 × 10^6^ cells/well with various concentrations of islatravir or cyclosporin A, a positive-control inhibitor, under 5% CO_2_ at 37 °C for 30 min. Uptake was then initiated by addition of probe substrates as previously described [58], with the exception that 0.1 µM [^3^H]pitavastatin was used as probe substrate for OATP1B1 and uptake time of OATP1B1 and OATP1B3 inhibition assays was 2 min. Pyrimethamine (5 μM) was used as a positive control inhibitor in MATE1 and MATE2K inhibition studies. Transporter-mediated uptake was calculated by subtracting the uptake rate in control cells from that in transporter-expressing cells. Data were normalized to % control, where uptake in the absence of test compound was 100%.

The inhibitory effect of islatravir (0.22–200 µM) on MDR1 P-gp-mediated bi-directional transport of [^3^H]-digoxin (0.1 µM) was assessed in LLC-PK1 cell lines stably expressing MDR1 P-gp, as previously described [55].

Inhibition of BCRP-mediated [^3^H]methotrexate uptake was assessed in BCRP-containing Sf9 membrane vesicles as previously described [58]. Briefly, [^3^H]methotrexate was mixed with BCRP vesicles (Thermo Fisher Scientific, Waltham, MA, USA) and various concentrations of islatravir or 10 µM Ko143, and preincubated at 37 °C for 5 min. Uptake was initiated by adding adenosine triphosphate (ATP) or adenosine monophosphate (AMP), followed by incubation at 37 °C for 5 min. Uptake was stopped and samples transferred to pre-wetted 96-well glass-fiber filter plates, and vacuum was applied. The washing steps and sample analysis were performed as previously described [58]. Similarly, inhibition of BSEP, MRP2-, MRP3-, and MRP4-mediated uptake of probe substrates [^3^H]taurocholic acid (1 µM), [^14^C]ethacrynic acid glutathione conjugate (1 µM), [^3^H]estradiol 17β-d-glucuronide (1 µM), and [^3^H]folic acid (10 µM), respectively, was evaluated in Sf9 membrane vesicles containing BSEP, MRP2, MRP3, or MRP4 (Thermo Fisher Scientific, Waltham, MA, USA), in the presence or absence of various concentrations of islatravir, or 100 µM atorvastatin (BSEP) or 100 µM bromosulfophthalein (MRP2, MRP3, or MRP4) as control inhibitors. Transporter-mediated uptake was calculated by subtracting the uptake in the presence of AMP from that in the presence of ATP, and data were normalized to percent control, where uptake in the absence of test compound was 100%.

### 2.6. Evaluation of Islatravir as a Victim of Drug–Drug Interactions via Transporters

To assess uptake in recombinant transporter expressing cell lines, uptake of 1 μM [^3^H]islatravir into MDCKII, MDCKII-OAT1, MDCKII-OAT3, CHO-K1, and CHO-K1-OCT2 cells and 2 μM [^14^C]islatravir into CHO-K1, CHO-K1-MATE1, MDCKII, and MDCKII-MATE2K cells was measured using the methods reported previously, with a cell density of 0.4 × 10^6^ cells/well [57]. [^3^H]*p*-aminohippuric acid (1 μM), [^3^H]estrone sulfate (1 μM), and [^14^C]tetraethylammonium (1 μM) were used as positive control substrates of OAT1, OAT3, and OCT2, respectively. [^14^C]Metformin (5 μM) was used as control substrate of MATE1 and MATE2K. Probenecid (1 mM) was used as control inhibitor of OAT1 and OAT3. Quinidine (0.1 mM) was used as control inhibitor of OCT2, and pyrimethamine (5 μM) as control inhibitor of MATE1 and MATE2K. Based on internal assay calibrations, and in line with regulatory agency recommendations [15,16], islatravir was considered a transporter substrate when uptake was time-dependent, inhibited by the control transporter inhibitor, and 1.5-fold higher in the transporter-expressing cell line compared with the control cell line, at a minimum of 2 time-points.

To study uptake in MDR1 P-gp-containing membrane vesicles, the time- and ATP-dependent uptake of [^14^C]islatravir was measured in control and MDR1 P-gp-containing Sf9 membrane vesicles (Thermo Fisher Scientific, Waltham, MA, USA) [57]. Briefly, [^14^C]islatravir (5 μM) or [^3^H]N-methylquinidine (0.5 μM), with or without cyclosporin A (10 µM) was pre-incubated with ATP-regenerating reagent or AMP reagent for 5 min at 37 °C. Uptake was initiated by the addition of substrate solution to MDR1 P-gp, or control vesicles, followed by incubation at 37 °C for 0–10 min. Uptake was stopped and samples transferred to a pre-wetted 96-well glass fiber filter plate, and vacuum was applied. The washing steps and sample analysis were performed as previously described [55,57]. Based on internal assay calibrations, and in line with regulatory agency recommendations [15,16], islatravir was considered a substrate of MDR1 P-gp when uptake was time-dependent, inhibited by the control transporter inhibitor cyclosporin A, and 1.5-fold higher in the presence of ATP compared with its absence, at a minimum of 2 time-points.

Bidirectional transport of islatravir (2 μM), with or without Ko143 (5 μM, a prototypic BCRP inhibitor), was measured across MDCKII and MDCKII-BCRP cell monolayers as previously described [55,57]. Prazosin (1 μM), with or without 5 μM Ko143, was used as the positive control. Samples were analyzed quantitatively by LC-MS/MS. The apparent permeability (P_app_) and efflux ratios were calculated as described below and as previously reported [57,59]. Based on internal assay calibrations, and in line with regulatory agency recommendations [15,16], islatravir was considered a substrate of BCRP when the fold-difference in efflux ratio between the control and BCRP expressing cells was >2 and inhibited by the control transporter inhibitor Ko143.

$$P_{\mathrm{app}}=\frac{\mathrm{Volume}\;\mathrm{of}\;\mathrm{Receptor}\;\mathrm{Chamber}\;\left(\mathrm{mL}\right)}{\left[\mathrm{Area}\;\mathrm{of}\;\mathrm{Membrane}\;\left({\mathrm{cm}}^{2}\right)\right]\left[\mathrm{Initial}\;\mathrm{Concentration}\;\left(\mu M\right)\right]}\times \frac{\Delta \;\mathrm{in}\;\mathrm{Concentration}\;\left(\mu M\right)}{\Delta \;\mathrm{in}\;\mathrm{Time}\;\left(s\right)}$$

$$Efflux\;ratio=\frac{P_{\mathrm{app}}\left(B\;\mathrm{to}\;A\right)}{P_{\mathrm{app}}\left(A\;\mathrm{to}\;B\right)}$$

## 3. Results

### 3.1. Islatravir Exhibited Low Plasma Protein Binding

The in vitro assessment indicated that the reversible binding of islatravir to proteins in human plasma was low, with mean unbound fractions in plasma of 0.96 ± 0.04 (±standard deviation) at 0.1 µM islatravir. Islatravir, at 0.1 µM, also exhibited low binding to plasma proteins from mouse, rat, rabbit, and monkey, with unbound fractions exceeding 0.84 across species. Islatravir binding to plasma proteins did not exhibit concentration dependence at concentrations ranging between 0.1 and 10 µM, for all species tested.

The partitioning of islatravir between human red blood cells and plasma was evaluated in vitro, with a mean blood:plasma concentration ratio of 1.3 ± 0.0 (±standard error) at 0.1 µM; islatravir did not exhibit concentration-dependent partitioning over the 0.01 to 10 µM concentration range.

### 3.2. Islatravir Was Metabolized by Adenosine Deaminase

In cryopreserved human hepatocyte incubations, no detectable metabolites of islatravir were observed; however, additional in vitro studies have shown that islatravir is extensively metabolized by ADA. Considering the high expression of ADA in the human intestine (compared with the liver) [60], this tissue was used to assess islatravir metabolism. In human intestinal S9 fraction, [^3^H]islatravir exhibited low turnover, with 4′-ethynyl-2-fluoro-2′-deoxyinosine (M4; Figure 1B) being the only metabolite detected by LC-MS/MS, interfaced with online radiometric detection. The ADA-mediated metabolism of islatravir was further studied in vitro by incubation with recombinant human ADA. In these studies, very slow ADA-dependent deamination to M4 was observed, which is consistent with previously reported results for islatravir [61]. Enzyme kinetics for recombinant human ADA showed that the rate of M4 formation increased linearly over the 1–250 µM islatravir concentration range, indicating that recombinant ADA has a relatively low affinity for islatravir (Michaelis constant [K_m_] >250 µM), consistent with ADA being a high-capacity enzyme.

### 3.3. Islatravir Was Partially Cleared via Renal Excretion in Nonclinical Species

In addition to ADA-mediated metabolism, nonclinical studies demonstrated renal clearance of islatravir to be 15.4, 11.5, 5.3, and 8.7 mL/min/kg in mouse, rat, rabbit, and monkey, respectively. The renal excretion of unchanged islatravir contributed 61%, 17%, 31%, and 51% of the total plasma clearance in mouse, rat, rabbit, and monkey models, respectively (Table 1). For all species tested, renal clearance exceeded the glomerular filtration rate, suggesting that in addition to filtration, renal clearance of islatravir is partially mediated by active transport in these species. Based on these findings, renal excretion of unchanged islatravir is anticipated to contribute to the overall elimination in humans.

### 3.4. Islatravir Did Not Inhibit or Induce Major Drug-Metabolizing Enzymes

The inhibitory effect of islatravir on CYP isoforms and UGT1A1 in pooled human liver microsomes is summarized in Table 2. No reversible inhibition was observed with islatravir on any CYP isoform tested (1A2, 2B6, 2C8, 2C9, 2C19, 2D6, or 3A4) at concentrations up to 100 μM, indicating a half maximal inhibitory concentration (IC_50_) greater than 100 μM for all reactions. Islatravir concentrations of up to 200 μM did not inhibit CYP3A4, indicating an IC_50_ greater than 200 μM. Pre-incubation of 10 and 50 µM islatravir for up to 30 min in human liver microsomes caused no time-dependent inhibition of CYP1A2, 2B6, 2C8, 2C9, 2C19, 2D6, and 3A4. No inhibition of UGT1A1-mediated estradiol 3-glucuronidation was observed with islatravir up to 100 μM in human liver microsomes, indicating an IC_50_ greater than 100 μM.

The potential for islatravir to induce CYP3A4, CYP2B6, or CYP1A2 was assessed in cryopreserved hepatocytes from three donors, after 48 h exposure to 0.1–20 μM islatravir. The mRNA response for CYP3A4, CYP2B6, or CYP1A2 was less than that of the vehicle control for each tested concentration of islatravir (Table 3), which was less than 20% of respective positive controls, indicating islatravir was not an inducer of these enzymes [14,15,16]. In line with these data, corresponding enzyme activity of CYP3A4, CYP2B6, or CYP1A2 in the same incubations was not greater than 1.1-fold of solvent control for all islatravir concentrations and CYP isoforms tested.

### 3.5. Islatravir Did Not Inhibit Major Hepatic Transporters at Clinically Relevant Concentrations

In recombinant cell lines, concentrations of islatravir of up to 300 µM did not inhibit the OATP1B1-, OATP1B3-, and OCT1-mediated uptake of pitavastatin, sulfobromophthalein, or metformin, respectively. Similarly, islatravir concentrations of up to 100 µM did not inhibit the BSEP-, MRP2-, MRP3-, and MRP4-mediated ATP-dependent uptake of taurocholic acid, ethacrynic acid glutathione conjugate, E_2_17βG, or folic acid, respectively, in Sf9 membrane vesicles containing these efflux transporters. This indicates IC_50_ values greater than 300 μM for OATP1B1, OATP1B3, and OCT1, and greater than 100 μM for the other hepatic transporters tested (Table 2).

### 3.6. Islatravir Did Not Inhibit Major Renal Transporters at Clinically Relevant Concentrations

OAT1-mediated cidofovir uptake in recombinant cell lines was not inhibited by concentrations of islatravir up to 100 μM, whereas islatravir inhibited OAT3-mediated estrone sulfate uptake and OCT2-mediated metformin uptake by 31% and 15% at 100 μM, respectively. Metformin uptake into recombinant cell lines expressing the renal efflux transporters MATE1 or MATE2K was not inhibited by concentrations of islatravir up to 75 µM. Taken together, these data indicate that the IC_50_ values for inhibition of the tested renal drug transporters are greater than 75 μM for MATE1 and MATE2K, and greater than 100 μM for OAT1, OAT3, and OCT2 (Table 2).

### 3.7. Islatravir Did Not Inhibit MDR1 P-gp and BCRP at Clinically Relevant Concentrations

**Figure 3:**
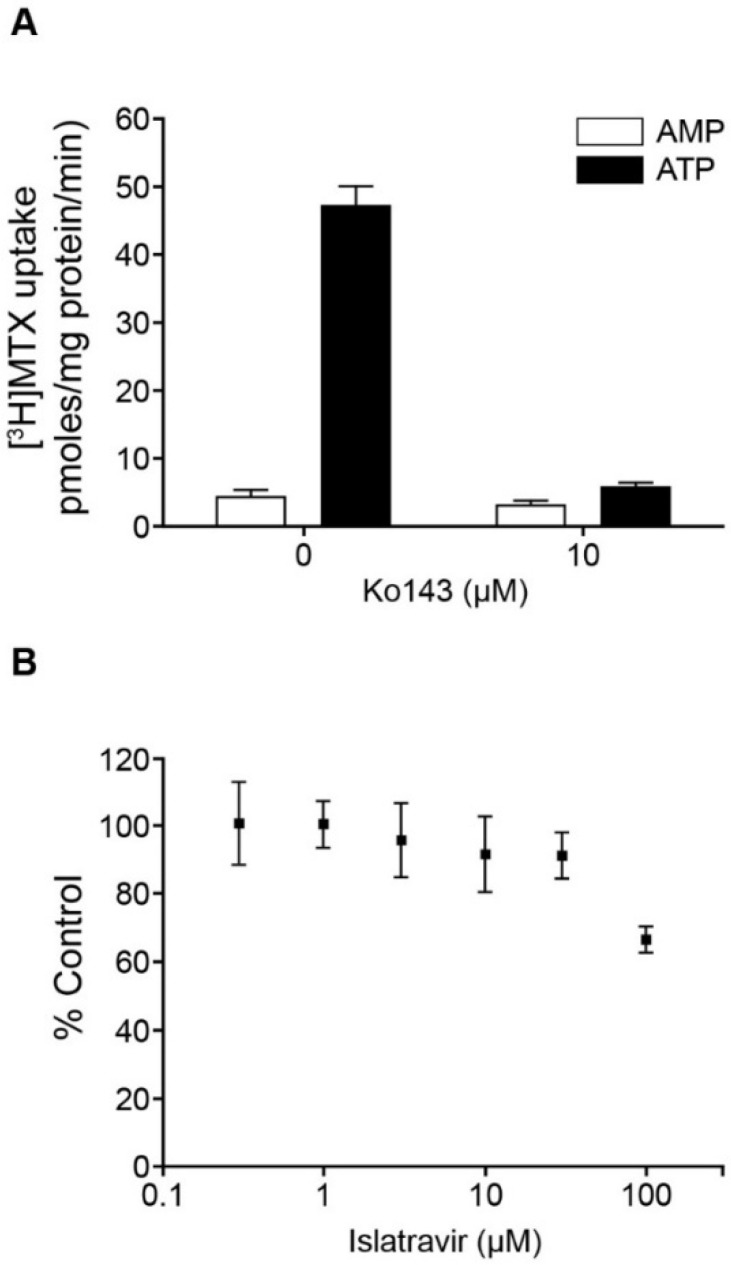
Inhibition of BCRP by islatravir. (**A**) Positive control, the effect of Ko143 (10 μM) on the uptake of [^3^H]methotrexate (MTX 10 μM) in BCRP-containing Sf9 membrane vesicles, in the presence of ATP or AMP. (**B**) The effect of islatravir on ATP-dependent uptake of [^3^H]methotrexate (10 μM) in BCRP-containing Sf9 membrane vesicles (percentage of control). The experiment was performed in triplicate. All data are mean ± SD. AMP, adenosine monophosphate; ATP, adenosine triphosphate; BCRP, breast cancer resistance protein; [^3^H]MTX, [^3^H]methotrexate; SD, standard deviation.

The potential for islatravir to inhibit the broadly expressed efflux transporters MDR1 P-gp and BCRP was assessed in vitro, in accordance with current regulatory guidance. Islatravir showed no concentration-dependent inhibition of MDR1 P-gp-mediated digoxin transport across monolayers of recombinant cells expressing this transporter, up to 200 μM, indicating an IC_50_ greater than 200 μM. In a membrane vesicle assay, islatravir inhibited 35% of human BCRP-mediated methotrexate uptake at 100 μM, indicating an IC_50_ greater than 100 μM (Figure 3).

### 3.8. Islatravir Was Not a Substrate of Major Renal Transporters

As islatravir is expected to be partially eliminated by urinary excretion in humans, the transport of islatravir via renal transporters OCT2, OAT1, OAT3, MATE1, and MATE2K was investigated in vitro (Table 4). Across the time points tested (1–5 min for OCT2, OAT1, and OAT3, and 5–20 min for MATE1 and MATE2K), uptake of islatravir into recombinant cell lines expressing these transporters at any time point was 0.7 to 1.3-fold compared with that in parental cells, indicating that islatravir was not a substrate of these renal transporters [15,16]. The assays were considered functional as the uptake of the positive control substrates was 6 to 161-fold higher in the transporter-expressing cell lines compared with control cell lines and was fully inhibited by the control transporter inhibitors.

### 3.9. Islatravir Was a Substrate of BCRP, but Not MDR1 P-gp

MDR1 P-gp and BCRP are widely expressed efflux transporters, and thus islatravir was investigated as a substrate of these transporters. Uptake of islatravir into membrane vesicles containing MDR1 P-gp was similar to that in control vesicles (3.7 ± 1.3 and 5.7 ± 2.1 pmole/mg protein (mean ± standard deviation), respectively in the presence of ATP at the final time point of 10 min) and was not ATP-dependent, indicating that islatravir was not a substrate of MDR1 P-gp [15,16]. The assay was considered functional as the uptake of the positive control substrate was 26-fold higher in the presence of ATP, compared with its absence, and was fully inhibited by the control transporter inhibitor.

**Figure 4:**
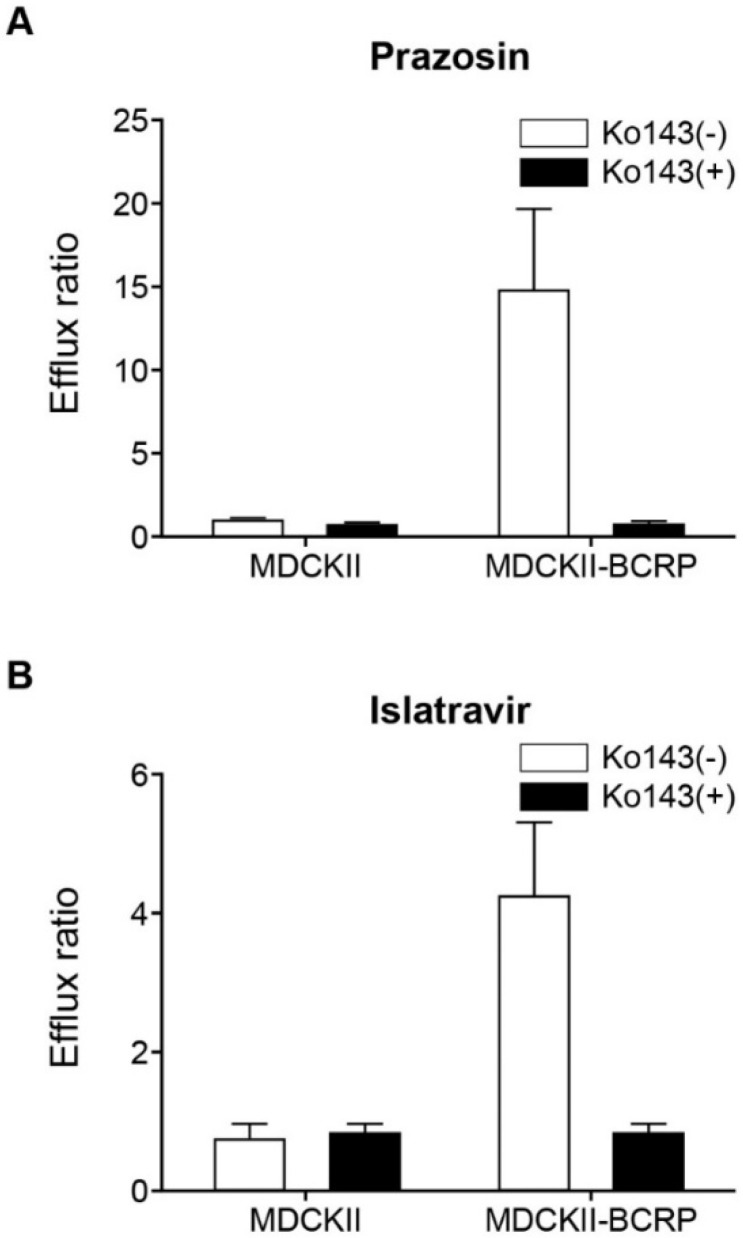
Bidirectional transport of islatravir across MDCKII and MDCKII-BCRP cell monolayers. (**A**) Positive control, efflux ratio of 1 μM prazosin across MDCKII and MDCKII BCRP cell monolayers, in the presence and absence of 5 μM Ko143 (control inhibitor). (**B**) Efflux ratio of 2 μM islatravir across MDCKII and MDCKII-BCRP cell monolayers after 3-h incubation, in the presence and absence of 5 μM Ko143. Efflux ratio: P_app_ (B to A)/P_app_ (A to B). The experiment was performed in triplicate, except for prazosin in MDCKII without Ko143 (*n* = 2). All data are mean ± SEM. BCRP, breast cancer resistance protein; SEM, standard error of the mean.

Islatravir was found to be a substrate of BCRP in a bi-directional transport assay, despite a relatively low apparent permeability coefficient (P_app_) of 1.6–2.2 × 10^6^ cm/s in MDCKII cells. The mean P_app_(B to A)/P_app_(A to B) efflux ratio for islatravir (2 μM) across BCRP-transfected monolayers was 4.3 ± 1.1 [mean ± standard error of the mean (SEM)], compared with a ratio of 0.8 ± 0.2 (mean ± SEM) for the untransfected control cells (Figure 4B). Furthermore, this BCRP-mediated transport of islatravir was inhibited by the addition of the prototypic BCRP inhibitor Ko143 (5 μM) with the efflux ratio reduced to 0.9 ± 0.1 (mean ± SEM). In comparison, the efflux ratio of the positive control substrate prazosin in BCRP-transfected monolayers was 14.9 ± 4.9 (mean ± SEM), compared with a ratio of 1.0 ± 0.09 (mean ± SEM) for the untransfected control cells (Figure 4A).

## 4. Discussion

The in vitro studies reported here characterize the potential drug interaction profile of islatravir, a novel NRTTI in clinical development for the treatment and prevention of HIV-1 [17,18]. The drug-metabolizing enzymes and transporters selected for evaluation were based upon the disposition of islatravir and the commonly prescribed medications expected to be taken concomitantly with islatravir (Figure 2), in line with current regulatory guidance and requirements [14,15,16].

Islatravir was found to have an equal distribution in blood and plasma and low binding to plasma proteins. First-pass metabolism is expected based on the abundance of ADA in the intestine [60]. In vitro assessment of the metabolism of islatravir in intestinal S9 fraction showed inefficient deamination, as observed in the presence of recombinant human ADA. The enzyme kinetics for recombinant human ADA showed a linear rate of M4 formation at concentrations of islatravir between 1 and 250 µM, which indicated that the ADA-catalyzed metabolism of islatravir to M4 is a high-capacity reaction, with a K_m_ greater than 250 µM. Thus, saturation of ADA-mediated metabolism is not expected at clinically relevant doses of islatravir. Previous studies have shown that the 2-fluoro group in the islatravir structure significantly decreases its susceptibility to hydrolysis by ADA, increasing its intracellular half-life [18,20,24]. There was no evidence of islatravir metabolism in human cryopreserved hepatocytes, suggesting that hepatic metabolism may not contribute significantly to the elimination of islatravir. Islatravir was, however, partially eliminated via urinary excretion in animal models and is expected to be the same in humans.

In the current in vitro analysis, probe drug substrates were used to assess islatravir as a potential perpetrator of metabolizing enzyme and/or transporter-mediated drug–drug interactions. The probe drugs used are known substrates of a given metabolic or transporter pathway [30,63]. The pathways by which these probe drugs are metabolized and transported are well established and any observed drug interaction can be applied across other more commonly prescribed agents, which are known to have the same metabolic or transport pathway.

In these studies, the potential interaction of islatravir with major drug-metabolizing enzymes, CYP isoforms, and UGT1A1, was assessed. The results demonstrate no reversible inhibition of CYP3A4 up to 200 μM islatravir, indicating an IC_50_ greater than 200 μM. For other CYP isoforms and UGT1A1, no reversible inhibition was shown at islatravir concentrations up to 100 μM, indicating IC_50_ values greater than 100 μM. These IC_50_ values are well above the expected therapeutic C_max_ of islatravir and exceed the projected C_max_ of 1.01 μM for a 60 mg oral dose by almost two orders of magnitude [36], indicating wide margins to any potential islatravir-mediated effects for doses up to, and including, 60 mg (Table 2).

Hepatic drug-metabolizing enzymes are associated with a large proportion of clinically relevant drug–drug interactions, with CYPs having a role in the metabolism of 70–80% of drugs [64]. Drugs commonly prescribed in PLWH metabolized by CYPs and UGT1A1 include the proton-pump inhibitor omeprazole, the antiplatelet drug clopidogrel, the selective serotonin reuptake inhibitor citalopram, the opioid buprenorphine, and the antibiotic rifampin, amongst others [30,37,38,39,43,44,45,47,48,49,51,52,53,65,66].

No time-dependent inhibition by islatravir was observed for CYP1A2, 2B6, 2C8, 2C9, 2C19, 2D6, and 3A4. CYP3A4 is the most abundantly expressed drug-metabolizing enzyme in humans most commonly associated with drug interactions. CYP3A4 is responsible for the metabolism of numerous drugs, including the benzodiazepine alprazolam, atorvastatin, antihistamines, and a majority of antiretroviral agents [30,63,66].

In addition to drug-metabolizing enzymes, drug transporters play an important role in drug distribution and elimination; thus, the impact of islatravir on major uptake and efflux transporters, and the effect of these transporters on islatravir, was assessed. Islatravir demonstrated no inhibitory effect on hepatic uptake transporters OATP1B1, OATP1B3, and OCT1, which are essential for the uptake of major drugs, such as statins and angiotensin II receptor blockers, from sinusoidal blood into the liver for clearance [67]. At the 60 mg dose, the projected maximum free concentration of islatravir at the liver inlet is approximately 10 μM, which is more than 30-fold lower than the maximum concentration of islatravir for which there was no inhibition of hepatic uptake transporters in these studies (Table 2). Cardiovascular disease and diabetes are increasing in prevalence in PLWH [2,7,8,30]; importantly, the commonly prescribed drugs to treat these conditions, including atorvastatin, rosuvastatin, angiotensin II receptor blockers, and metformin, which are hepatic uptake transporter substrates, are not anticipated to interact with islatravir. Islatravir also demonstrated no inhibitory effect on the hepatic efflux transporters BSEP, MRP2, MRP3, and MRP4, which are involved in the hepatic efflux of endogenous bile acids [67,68]. Inhibition of these transporters, particularly BSEP, is associated with drug-induced liver injury and cholestasis [33,69].

Considering the anticipated contribution of renal excretion in the elimination of islatravir in humans, the lack of metabolism of islatravir observed in human hepatocytes, and the low expression of ADA in the liver [60], hepatic metabolism is not expected to be a significant route of elimination; therefore, islatravir was not assessed as a substrate of hepatic drug-metabolizing enzymes or uptake transporters.

Renal uptake transporters, including OAT1, OAT3, and OCT2, are involved in the elimination of commonly prescribed medications, such as metformin, antiarrhythmics, and diuretics, as well as multiple antibiotics and antiviral drugs, such as adefovir, ganciclovir, and tenofovir [30,70]. Tenofovir disoproxil fumarate is a nucleoside reverse transcriptase inhibitor that is metabolized by plasma and tissue esterases to tenofovir [71], which is actively transported by OAT1 and OAT3 into renal proximal tubule cells and then eliminated into the urine by MRP2 and MRP4. Inhibition of these transporters may lead to drug accumulation and renal toxicity [72]. At clinically relevant concentrations, islatravir did not inhibit OAT1, OAT3, or OCT2, with IC_50_ values greater than 100 µM. Furthermore, islatravir was not found to be a substrate of these transporters. In addition, islatravir was neither a substrate nor an inhibitor of the renal efflux transporters MATE1, MATE2K, and MDR1 P-gp. This finding indicates that islatravir is not likely to be either the perpetrator or victim of renal transporter-based drug–drug interactions with renal uptake substrates or inhibitors, such as the HIV integrase strand transfer inhibitor dolutegravir and the histamine-2 receptor antagonist cimetidine [30,70]. The IC_50_ values for the interactions between islatravir and major renal transporters exceed the projected maximum unbound plasma concentrations for a 60 mg dose by approximately 100-fold [73], indicating wide margins for dosing without the consideration for drug–drug interactions (Table 2).

Islatravir was not found to be an inhibitor of BCRP at clinically meaningful concentrations (Table 2); however, it was found to be a substrate of BCRP in vitro (Figure 3). Unlike other substrates of BCRP such as rosuvastatin and sulfasalazine [32], islatravir is unlikely to be the victim of BCRP-mediated drug-drug interactions due to its good absorption in vivo, and an anticipated lack of major hepatic secretory clearance [26,74]. Should BCRP contribute to the intestinal efflux of islatravir in vivo, co-administration of an inhibitor of BCRP would only serve to increase absorption of islatravir, which is already well absorbed and is expected to have a favorable drug–drug interaction and toxicity profile [26,74].

Together, these findings are in good agreement with clinical studies conducted to date that demonstrated a lack of drug–drug interactions between islatravir and other agents in participants without HIV. A PK and safety study of islatravir co-administered with doravirine, which is primarily metabolized by CYP3A4, demonstrated no clinically meaningful effects on the PK of either drug [54,75]. Another PK and safety study demonstrated no meaningful drug–drug interactions between islatravir and tenofovir disoproxil fumarate, which is eliminated renally via OAT1 and OAT3, and dolutegravir, which is hepatically metabolized by UGT enzymes and CYP3A4 [70,71,76]. No significant drug–drug interactions have been observed following co-administration of islatravir with levonorgestrel/ethinyl estradiol [77], common components of hormonal contraceptives that are extensively metabolized by CYP3A4, are glucuronidated, and undergo biliary and urinary excretion [78].

Due to its high potency and long intracellular half-life, islatravir remains efficacious at very low doses. Combined with its lack of inhibition of major metabolizing enzymes and drug transporters, islatravir has low potential for drug–drug interactions. Using static drug–drug interaction risk assessment models based on regulatory agency guidelines, islatravir is considered at low risk of drug–drug interactions with major drug transporters and drug-metabolizing enzymes due to the low exposures at therapeutic doses and the lack of inhibition observed in vitro [14,15,79] (Table 2).

## 5. Conclusions

The lack of interaction of islatravir with major drug-metabolizing enzymes and drug transporters and their substrates reinforces the favorable drug–drug interaction profile of islatravir and its potential to be administered as part of combination ART and alongside concomitant medications. This finding is of particular clinical relevance for PLWH who may require polypharmacy for the management of both HIV and common comorbidities, such as diabetes, cardiovascular disease, and depression. Islatravir is not expected to interact with the major pathways associated with other antiretroviral agents, including dolutegravir, doravirine, and tenofovir disoproxil fumarate [54,71,76] as well as with commonly prescribed medications, including metformin, omeprazole, clopidogrel, statins, alprazolam, buprenorphine/naloxone, selective serotonin reuptake inhibitors, oral contraceptives, and rifampin [77]. These results support the continued clinical evaluation of islatravir as an option across diverse populations for the treatment and prevention of HIV-1 infection.

## Figures and Tables

**Table 1:** Renal clearance and excretion in nonclinical species.

Species	Renal Clearance (mL/min/kg)	Renal Excretion (% Total Plasma Clearance)
Mouse	15.4	61
Rat	11.5	17
Rabbit	5.3	31
Rhesus macaque	8.7	51

**Table 2:** Drug–drug interaction risk calculation for islatravir.

**Islatravir Risk of Interaction with Metabolic Enzymes**					
**Enzyme**	**Mechanism of Inhibition**	**Islatravir IC_50_ (µM) ^a^**	**Maximum Unbound Plasma Concentration ^b^ (I_max,u_) to K_i,u_^c^ Ratio (µM)**	**Intestinal Concentration ^d^ (I_gut_) to K_i,u_^c^ Ratio (µM)**	**DDI Potential ^f^**
CYP1A2, 2B6, 2C8, 2C9, 2C19, 2D6	Reversible	>100	<0.019	N/A	Low risk
CYP3A4	Reversible	>200	<0.010	<8.2	Low risk
UGT1A1	Reversible	>100	N/A	<16.4	Low risk ^g^
CYP1A2, 2B6, 2C8, 2C9, 2C19, 2D6, 3A4	Time dependent	>50	N/A	N/A	Low risk ^h^
**Islatravir Risk of Interaction with Drug Transporters**					
**Transporter**	**Islatravir IC_50_ (µM) ^a^**	**Maximum Unbound Plasma Concentration ^b^ (I_max,u_) to IC_50_ Ratio (µM)**	**Intestinal Concentration ^d^ (I_gut_) to IC_50_ Ratio (µM)**	**Maximum Unbound Inlet Concentration ^e^ (I_in,max,u_) to IC_50_ Ratio**	**DDI Potential ^f^**
OATP1B1, OATP1B3, OCT1	>300	N/A	N/A	<0.035	Low risk
OAT1, OAT3, OCT2	>100	<0.010	N/A	N/A	Low risk
MATE1, MATE2K	>75	<0.013	N/A	N/A	Low risk
BCRP	>100	<0.010	<8.2	N/A	Low risk
MDR1 P-gp	>200	<0.005	<4.1	N/A	Low risk

^a^ When either no inhibition or <50% inhibition was observed at the highest concentration tested, IC_50_ was assumed to be above the highest concentration tested, for the purpose of risk assessment. Maximum unbound plasma concentration, intestinal concentration, and unbound inlet concentration to IC_50_ ratios are predicted based on the parameters and calculations below: ^b^ I_max,u_ = C_max_ * f_u.p_ where C_max_ = 1.01 µM for 60 mg at steady state and f_u.p_ = 0.96. ^c^ K_i,u_ calculated as IC_50_/2 * fu,mic, assuming competitive inhibition, assuming worst case scenario with a K_i_ of 50 µM and a calculated fu,mic = 1 [62]. ^d^ I_gut_ = 60 mg islatravir dose/250 mL = 818 µM. ^e^ I_in,max,u_ = f_u,b_ * (I_max,b_ + ((F_a_F_g_ × k_a_ × Dose)/Q_h_)) where f_u,b_ = f_u,p_/R_B_, I_max,b_ = C_max_ * R_B_, R_B_ = 1.3, Q_h_ = 1617 mL/min, and assumes F_a_F_g_ = 1 and k_a_ = 0.1 min^−1^. ^f^ Risk assessment based on guidance provided by FDA, EMA, and PMDA [14,16,30]. ^g^ Because no inhibition of UGT1A1 was observed at 100 µM, the IC_50_ is considered to be significantly higher than 100 µM, and thus the I_gut_ to K_i,u_ ratio of <16.4 is conservative and the potential for interaction at the gut level is considered to be low. ^h^ Because time-dependent inhibition was not observed, determination of k_inact_ and K_i_ was not warranted, precluding the need for further risk assessment as outlined by agency guidance. N/A: Indicates calculations are not relevant for transporter or enzyme location. BCRP, breast cancer resistance protein; C_max_, maximum plasma concentration; CYP, cytochrome P450; DDI, drug–drug interaction; EMA, European Medicines Agency; FDA, Food and Drug Administration; F_a_, fraction absorbed; F_g_, intestinal availability; f_u.p_, unbound fraction in plasma; IC_50_, half maximal inhibitory concentration; I_gut_, intestinal luminal concentration; I_in,max,u_, estimated maximum plasma inhibitor concentration at the liver inlet; I_max,u_, maximal unbound plasma concentration; k_a_, absorption rate constant; K_i_, inhibition constant; MATE, multidrug and toxin extrusion protein; MDR1 P-gp, multidrug resistance protein 1 P-glycoprotein; OAT, organic anion transporter; OATP, organic anion transporting polypeptide; OCT, organic cation transporter; PMDA, Pharmaceuticals and Medical Devices Agency; Q_h_, hepatic blood flow rate; R_B_, blood-to-plasma ratio; TDI, time-dependent inhibition; UGT1A1, uridine diphosphate glucuronosyltransferase 1A1.

**Table 3:** Effect of islatravir on CYP mRNA in human hepatocytes.

Concentration [µM]		mRNA Mean Fold Change ± SD ^a^		
		CYP3A4	CYP2B6	CYP1A2
DMSO (vehicle)	NA	1.0 ± 0.0	1.0 ± 0.0	1.0 ± 0.0
Rifampin (control)	10	9.9 ± 2.7	ND	ND
Phenobarbitol (control)	1000	ND	18.5 ± 1.9	ND
Omeprazole (control)	50	ND	ND	26.4 ± 8.6
Islatravir	0.1	0.6 ± 0.2	0.5 ± 0.1	0.4 ± 0.2
	0.5	0.6 ± 0.2	0.5 ± 0.2	0.4 ± 0.2
	1	0.6 ± 0.2	0.7 ± 0.2	0.5 ± 0.3
	5	0.5 ± 0.1	0.7 ± 0.1	0.4 ± 0.3
	10	0.6 ± 0.1	0.9 ± 0.3	0.5 ± 0.4
	20	0.1 ± 0.1	0.4 ± 0.3	0.2 ± 0.2

^a^ Mean ± SD fold change was calculated by dividing mRNA levels in treated samples, by those in the DMSO vehicle control samples, for *n* = 3 donors. Fold change for vehicle control was set to 1.0 CYP, cytochrome P450; DMSO, dimethylsulfoxide; NA, not applicable; ND, not determined; SD, standard deviation.

**Table 4:** Assessment of islatravir as a substrate of renal drug transporters in vitro.

Transporter	Islatravir Uptake ^a^ (pmole/10^6^ Cells)		Fold-Difference ^b^	Conclusions
	Control Cells	Transporter-Expressing Cells		
OCT2	0.97 ± 0.01	0.79 ± 0.14	0.81	Non-substrate
OAT1	0.69 ± 0.07	0.72 ± 0.04	1.04	Non-substrate
OAT3	0.69 ± 0.07	0.85 ± 0.06	1.23	Non-substrate
MATE1	2.90 ± 0.27	2.94 ± 0.20	1.01	Non-substrate
MATE2K	3.12 ± 0.17	3.56 ± 0.17	1.14	Non-substrate

^a^ Mean ± SD, *n* = 3 at last time point tested (5 min for OCT2, OAT1, OAT3, 10 min for MDR1 P-gp, and 20 min for MATE1 and MATE2K); ^b^ Fold-difference represents ratio of uptake into transporter-expressing cells to control cells; MATE, multidrug and toxin extrusion protein; OAT, organic anion transporter; OCT, organic cation transporter; SD, standard deviation.

## Data Availability

Available data can be obtained by contacting the corresponding author. Any data that are reasonably requested will be made available in a timely fashion to members of the scientific community with as few restrictions as feasible for noncommercial purposes. Subject to requirements or limitations imposed by local and/or U.S. government laws and regulations, we will make every effort to provide to those that request it additional information on the sources of materials used in the studies described here.
